# The impact of varicocelectomy on sperm DNA fragmentation and pregnancy rate in subfertile men with normal semen parameters: A pilot study

**DOI:** 10.1080/2090598X.2021.1889746

**Published:** 2021-02-14

**Authors:** Atef Fathi, Omar Mohamed, Osama Mahmoud, Gamal a Alsagheer, A.M. Reyad, Ahmed Abolyosr, Mohamed Sayed Abdel-Kader, Mohammed Saber-Khalaf

**Affiliations:** a Department of Urology, Qena Faculty of Medicine, South Valley University, Qina, Egypt; b Department of Urology, Sohag University Hospital, Sohag University, Sohag, Egypt

**Keywords:** Varicocelectomy, sperm DNA fragmentation, normal semen parameters, male subfertility

## Abstract

**Objective**: To evaluate the outcome of microscopic subinguinal varicocelectomy on sperm DNA fragmentation (SDF) and pregnancy rate in men with normal semen parameters.

**Patients and methods**: A pilot study that included male patients with a minimum of a 1-year history of male subfertility, normal semen parameters, a high percentage of SDF, and clinically palpable varicoceles. Microscopic subinguinal varicocelectomy was carried out for 45 patients (study group), while 40 patients had no intervention (control group). Semen analysis and SDF were measured before and at 6 months after the varicocelectomy. The pregnancy rate was assessed at the 6- and 12-month follow-ups.

**Results**: Between July 2014 and January 2019, 85 subfertile men were included in the study and completed 12 months of follow-up. The two groups were comparable in terms of their age, body mass index, infertility duration, infertility type, varicoceles laterality, and varicoceles grade (*P* values = 0.84, 0.34. 0.35, 1, 0.39, and 0.46, respectively). At 6 months after varicocelectomy, the mean SDF was reduced in both groups, and this reduction was statistically higher in the varicocelectomy group (*P* < 0.001). After 1-year, spontaneous pregnancy was achieved in 62% of the patients in the varicocelectomy group compared to 30% in the control group (*P* = 0.009).

**Conclusion**: Varicocelectomy has a positive impact on SDF and spontaneous pregnancy in infertile men with clinically palpable varicoceles and normal semen parameters.

**Abbreviations**: BMI: body mass index; DFI: DNA fragmentation Index; SDF: sperm DNA fragmentation

## Introduction

Varicoceles are an abnormal dilatation of the pampiniform plexus, which is the venous drainage of the testicle. The prevalence of varicoceles is estimated to be 45% of primary male infertility patients and up to 80% among males with secondary infertility [1]. Surgical treatment of varicocele has a significant effect on semen parameters, so it is considered the ‘gold standard’ treatment for subfertile males with palpable varicoceles associated with abnormal semen analysis [2].

Semen analysis is an important infertility test for infertile couples. However, the 2010 WHO reference limits were defined according to the statistical fifth centile value for each semen parameter. Thus, it is important to understand that the WHO reference limits should not be used as a strict indicator for male fertility, and more complex tests should be used especially in patients with idiopathic infertility [3]. Sperm DNA fragmentation (SDF), which is the percentage of spermatozoa with denatured DNA, is an important parameter that might contribute to male subfertility [4]. SDF, which is measured by the percentage of DNA fragmentation (DNA fragmentation index [DFI]), is more common in subfertile men than fertile men [5]. Recent studies proved the effect of SDF and oxidative stress on varicoceles-related subfertility. Loss of DNA integrity leads to arrest of embryo development, implantation, and early pregnancy loss [6].

Varicoceles induce a disturbance between the antioxidant and reactive oxygen species production leading to damage of sperm DNA and subsequent loss of normal embryo development [7]. Therefore, there is evidence supporting a beneficial role of varicocelectomy for subfertile males with abnormal semen parameters associated with high SDF [8]. Several studies in the last 10 years have addressed the effect of varicocelectomy on SDF and fertility, the majority of these studies included patients with palpable varicoceles and abnormal semen parameters according to WHO reference limits [5,9].

Our present study aimed to evaluate the outcome of microsurgical subinguinal varicocelectomy in terms of the spontaneous pregnancy rate and changes in the DFI in those patients. The study outcome measures were the changes in DFI%, which were measured before varicocelectomy and at 6 months after the varicocelectomy, and the spontaneous pregnancy rates at the 6- and 12-month follow-ups.

## Patients and methods

### Patients

This is a pilot study that was conducted at our tertiary centre between July 2014 and January 2019. Eligibility criteria for the study were that patients with at least a 1-year history of male factor subfertility, a clinically palpable varicocele associated with normal standard semen parameters according to the 2010 WHO criteria [10], and a high DFI% of >25% [7,11]. Female partners were evaluated by the female reproductive unit in our centre, and they were considered normal if they have normal menstruation, normal ovarian reserve, and negative hysterosalpingography. Patients with subclinical varicoceles, associated female factor fertility, recurrent varicoceles, cryptorchidism, genital infection, pyospermia, obesity, cigarette smoking habit, alcohol or drug abuse, patients exposed to gonadotoxins, or patients with cancer were excluded from the study. After explaining the procedure and the possible risks and benefits, 45 patients chose to proceed with varicocelectomy (study group), while 50 patients preferred to continue on follow-up without any interventions (control group). Both groups were advised about the importance of maintaining a good lifestyle, avoiding hot baths and tight clothes.

### Sample handling and SDF measurements

Two to three semen samples were provided by the patients after 3–5 days of sexual abstinence, and a standard semen analysis was performed within 1 h after collection according to the WHO 2010 guidelines. Those with abnormal semen analyses were excluded from the study. Another semen sample was obtained 6-months after varicocelectomy. Part of the sample was used for the measurement of SDF using the sperm chromatin dispersion, which is known as the Halo test [12]. The Halo test is based on the principle that sperms with fragmented DNA fail to produce the characteristic halo of dispersed DNA loops that is observed in sperm with unfragmented DNA, following acid denaturation and removal of nuclear proteins. This was confirmed by the analysis of DNA fragmentation using the specific DNA breakage detection-fluorescence *in situ* hybridisation. The percentage of spermatozoa with abnormal chromatin structure is represented by the DFI%, which is the ratio of single-stranded (denatured) DNA to the total DNA. The semen samples were all analysed by the same embryologist.

### Patients’ management

Microsurgical subinguinal varicocelectomy was successfully performed under spinal anaesthesia. All procedures were performed by a single surgeon using a Karl Zeiss operating microscope with a magnification of ×10–20, using the standard surgical technique [13].

Follow-up was conducted by reviewing the semen analysis results, including the DFI% at the 6-month follow-up, and also by a chart review and telephone calls at the 6- and the 12-month follow-ups.

The study was approved by the Institutional Review Board of South Valley University and conforms to the provisions of the Declaration of Helsinki. Written informed consent was obtained from the included patients before the study.

### Statistical analysis

The statistical analysis was carried out with the Statistical Package for the Social Sciences (SPSS®), version 26 (IBM Corp., Armonk, NY, USA). Frequency tables with percentages were used for categorical variables and for descriptive statistics the mean (SD) was used for numerical variables. A paired *t*-test was used for the analysis of the changes in DFI% before and after the intervention. An independent *t*-test was used to compare the changes in DFI% and pregnancy rate across the two groups. A chi-square test was used to compare the nominal data between the two groups. A *P* < 0.05 was considered statistically significant.

## Results

A total of 95 male patients with a 1-year history of male subfertility were recruited for the study. A total of 10 patients did not complete the follow-up in the control group. Thus, the study included 45 patients who underwent microsurgical subinguinal varicocelectomy, and 40 patients were on the follow-up (control group). All 85 patients completed the 1-year minimum follow-up period. The mean (SD) age of the patients and their wives in the study group was 33.4 (5.9) and 25.8 (4.4) years, respectively. The mean (SD) age of the patients and their wives in the control group was 33.1 (6) and 25.9 (3.59) years, respectively (*P* values = 0.84 and 0.99, respectively). There were no statistically significant differences between the two groups in terms of their body mass index (BMI), infertility duration, infertility type, varicoceles laterality, and varicoceles grade (*P* values = 0.34. 0.35, 1, 0.39, and 0.46, respectively). Most of the patients presented with primary infertility, which was ≥80% in both groups. The mean (SD) baseline sperm parameters were: sperm concentration 26.2 (8.9) millions/mL, percentage of progressive motility 33.4 (1.3)%, and sperm normal morphology 4.3 (0.2)%.

In the initial evaluation, the mean SDF was high being above the cut-off value of 25%, but was comparable between the two groups (*P* = 0.77). At the 6-month follow-up, the mean SDF was significantly reduced compared to the initial values (*P* = 0.044 and *P* < 0.001 in the control group and the study groups, respectively). On comparing the postoperative SDF% after 6 months, it was significantly lower in the varicocelectomy group, at a mean (SD) of 9.17 (4.63) vs 4 (4.51)% (*P*< 0.001). In the varicocelectomy group, all patients had a reduction in SDF, of whom 27 (60%) had a reduction in their SDF to <25%. In the control group, 20 (50%) patients had an improvement in SDF; however, no patients reduced their SDF to less than the cut-off value.

Although the pregnancy rate was higher in the varicocelectomy groups at the 6-month follow-up, the difference was statistically insignificant (*P* = 0.1). After 1 year, the pregnancy rate was 62.2% in the varicocelectomy group, which was nearly double the control group at 30% (*P* = 0.009). All of those patients had a spontaneous pregnancy with no miscarriage or a live birth. In the varicocelectomy group, spontaneous pregnancy was achieved in 85% of patients with a SDF of <25% compared to 27.8% in patients with a SDF of >25% (Table 1).

**Table 1.** Comparison of the baseline data and outcomes between the groups

Variable	Control group (40 patients)	Varicocelectomy group (45 patients)	*P*
Patient age, years, mean (SD)	33.13 (6)	33.42 (5.94)	0.84
Wife age, years, mean (SD)	25.9 (3.59)	25.88 (4.43)	0.99
BMI, kg/m^2^, mean (SD)	26.3 (2.52)	26.88 (2.73)	0.34
Infertility duration, months, mean (SD)	21.1 (7.25)	22.8 (7.98)	0.35
Varicocele, *n* (%) Unilateral Bilateral	8 (26.7) 22 (73.3)	8 (17.4) 37 (82.22)	0.39
Infertility, *n* (%) Primary Secondary	24 (80) 6 (20)	37 (82.22) 8 (17.4)	1
Varicocele grade, *n* (%) 2 3	13 (43.3) 17 (56.7)	15 (33.3) 30 (66.7)	0.46
DFI, %, mean (SD)	35.33 (6.12)	34.93 (5.56)	0.77
DFI at 6-month follow-up, %, mean (SD)	31.26 (5.34)	25.75 (5.15)	<0.001
Pregnancies at 0–6 months, *n* (%)	4 (13.3)	14 (31.1)	0.1
Total pregnancies at 1 year, *n* (%)	9 (30)	28 (62.2)	0.009

Regarding the sperm parameters at the 6-month follow-up, varicocelectomy improved the sperm count, the percentage of progressive motility, and the percentage of normal forms. Although the improvement in sperm count was statistically significant (*P* = 0.002), the improvement in the percentage of progressive motility and normal forms were statistically insignificant (*P* values 0.82 and 0.09, respectively; Table 2).

**Table 2.** Semen variables before and at 6 months after the varicocelectomy

Variable	Baseline (*N*= 45)	6-month follow-up (*N* = 45)	*P*
Sperm concentration, millions/mL, mean (SD)	26.1 (8.5)	32.5 (8.6)	0.002
Sperm progressive motility, %, mean (SD)	33.9 (1.6)	36.1.2 (6.3)	0.82
Sperm normal forms, %, mean (SD)	4.3 (0.5)	5.2 (1.8)	0.09

## Discussion

The present study demonstrated a positive effect of microsurgical subinguinal varicocelectomy on the reduction of SDF in subfertile men with clinically palpable varicoceles and normal semen parameters. The reduction of SDF was accompanied by a further improvement in the chance of spontaneous pregnancy, which was statistically significantly higher in the varicocelectomy group. The rate of spontaneous pregnancy achieved after varicocelectomy was double that achieved with medical treatment.

Despite the importance of semen analysis in the evaluation of male subfertility, it should be used with caution as previous studies reported a weak or no association between the semen parameters and the chance of first pregnancy [14–16]. It is worth mentioning that the WHO reference limits for sperm parameters do not reflect the normal values. The 1999 WHO reference limits were derived from experts’ experiences, while the WHO 2010 reference limits represent the statistical fifth centile of sperm parameters derived from an international population of fertile men. Measurement of sperm SDF has gained in importance for the evaluation of male subfertility over the past few years for the following reasons; firstly, some studies reported a strong correlation between SDF and time to pregnancy, they proved that men with subfertility have a higher DFI than fertile men [17,18]. Secondly, SDF does not affect only the time to natural pregnancy, but it has a negative effect on the outcome of intracytoplasmic sperm injection [18,19]. Finally, sperm chromatin abnormalities, which may be present in men with normal semen parameters, could not be evaluated by the routine semen analysis [20]. Thus, more complex tests like SDF are needed particularly in men with unexplained infertility. Nevertheless, we should emphasise that the cut-off value for SDF varies between studies. Infertility problems may occur when the SDF exceeds 20–25% [7]. In our present series, we used a cut-off value of 25%.

In 2013, Simon et al. [21] reported that 87% of patients with unexplained infertility have a high SDF of >25%, which was the cut-off value he used to compare fertile and infertile men. Varicoceles are associated with higher levels of SDF. In 2006, Smith et al. [22] reported a DFI% of 49% and 58% in patients with varicoceles with normal and abnormal semen parameters, respectively. In tandem with that, Peluso et al. [4] reported a higher level of chromatin abnormalities in patients with varicocele even with a normal semen profile. Thus, they recommended SDF measurement as a routine test for infertile men with varicocele.

In the present study, microsurgical subinguinal varicocelectomy statistically reduced the SDF to almost normal values. Also, sperm count, percentage of progressive motility, and percentage of normal forms increased. Consequently, spontaneous pregnancy was achieved in 62% of our present patients within 12 months of their varicocelectomy. In 2010, Smit et al. [8] found that varicocelectomy statistically reduced the SDF, and this reduction was more evident in the group who achieved normal pregnancy compared to those who remained subfertile. In 2014, Ni et al. [23] observed a high SDF in the subfertile group compared to the control group, and there was a statistically significant reduction in SDF after varicocelectomy, especially in those who achieved normal pregnancy. In 2018, Roque et al. [5] evaluated the relationship between elevated SDF and varicocele, and reported increased SDF in men with clinical varicocele. Thus, that study recommended varicocelectomy for subfertile men with palpable varicoceles and claimed that the repair of varicoceles had a beneficial effect on the chance of natural and assisted pregnancy. In addition to that, two recent trials documented the same beneficial effect of varicocelectomy on improving SDF and spontaneous pregnancy [24,25]. The improvement in SDF after varicocelectomy was linked to the higher grades of varicocele [26].

Several studies in the literature documented the beneficial impact of varicocelectomy on the reduction of SDF and an improvement in the rate of spontaneous pregnancy, all those studies excluded infertile men with normal semen parameters. In our present study, we analysed the effect of varicocelectomy on male subfertility patients with normal semen analysis but high SDF. The present study included a control group and assessed the outcome of varicocelectomy by the improvement in the SDF and the achievement of spontaneous pregnancy. However, no study goes without limitation. Our present study is a pilot study with a small sample size and a non-randomised control group. We recommend randomised controlled trials with a larger sample size to confirm our present results.

## Conclusion

Microsurgical varicocelectomy appears to have a beneficial role in reducing the percentage of SDF, increasing sperm parameters and the chance of spontaneous pregnancy. The improvement was significantly greater than that of the control group. Varicocelectomy could be offered as a treatment option for subfertile men with clinically palpable varicoceles and normal semen parameters if they have a high SDF. Randomised controlled trials are required to confirm our present results.
